# Deep CT to MR Synthesis Using Paired and Unpaired Data

**DOI:** 10.3390/s19102361

**Published:** 2019-05-22

**Authors:** Cheng-Bin Jin, Hakil Kim, Mingjie Liu, Wonmo Jung, Seongsu Joo, Eunsik Park, Young Saem Ahn, In Ho Han, Jae Il Lee, Xuenan Cui

**Affiliations:** 1 School of Information and Communication Engineering, INHA University, Incheon 22212, Korea; chengbinjin@inha.edu (C.-B.J.); liumj@inha.edu (M.L.); xncui@inha.ac.kr (X.C.); 2 Acupuncture and Meridian Science Research Center, Kyung Hee University, Seoul 02447, Korea; croquies@naver.com; 3 Team Elysium Inc., Seoul 93525, Korea; seongsu.joo@teamelysium.kr (S.J.); espark@teamelysium.kr (E.P.); 4 Department of Computer Engineering, INHA University, Incheon 22212, Korea; ahnsaem90@gmail.com; 5 Department of Neurosurgery, Pusan National University Hospital, Pusan 49241, Korea; farlateral@hanmail.net (I.H.H.); medifirst@pusan.ac.kr (J.I.L.)

**Keywords:** MR image synthesis, paired and unpaired training, generative adversarial networks, dual cycle-consistent loss, CT-based radiotherapy

## Abstract

Magnetic resonance (MR) imaging plays a highly important role in radiotherapy treatment planning for the segmentation of tumor volumes and organs. However, the use of MR is limited, owing to its high cost and the increased use of metal implants for patients. This study is aimed towards patients who are contraindicated owing to claustrophobia and cardiac pacemakers, and many scenarios in which only computed tomography (CT) images are available, such as emergencies, situations lacking an MR scanner, and situations in which the cost of obtaining an MR scan is prohibitive. From medical practice, our approach can be adopted as a screening method by radiologists to observe abnormal anatomical lesions in certain diseases that are difficult to diagnose by CT. The proposed approach can estimate an MR image based on a CT image using paired and unpaired training data. In contrast to existing synthetic methods for medical imaging, which depend on sparse pairwise-aligned data or plentiful unpaired data, the proposed approach alleviates the rigid registration of paired training, and overcomes the context-misalignment problem of unpaired training. A generative adversarial network was trained to transform two-dimensional (2D) brain CT image slices into 2D brain MR image slices, combining the adversarial, dual cycle-consistent, and voxel-wise losses. Qualitative and quantitative comparisons against independent paired and unpaired training methods demonstrated the superiority of our approach.

## 1. Introduction

Computed tomography (CT)-based radiotherapy [1] is currently used in radiotherapy planning and is reasonably effective. However, magnetic resonance (MR) imaging delivers superior contrast of soft tissue compared with the CT scans [2]; therefore, radiotherapy devices using MR imaging [3] are being developed. In addition, MR imaging is excellent for detecting very slight differences in soft tissues, ligaments, or organs.

However, compared with CT scans, MR imaging is not only much more expensive but also takes about six times longer: for example, for an MR imaging that takes about 30 min, the CT scan can be completed within 5 min. In addition, while the procedure for MR imaging is contraindicated for some patients due to claustrophobia, cardiac pacemakers, and artificial joints. Moreover, some patients cannot afford or do not have access to MR imaging due to the high cost.

Despite the many advantages of MR imaging, CT scans are much more widely used for diagnostic and therapeutic purposes across various clinical applications due to the abovementioned drawbacks. Moreover, it can be clinically desirable to infer an MR image from a CT scan for patients who are contraindicated because of claustrophobia, cardiac pacemakers, and artificial joints. Therefore, estimating a magnetic resonance imaging (MRI) from a CT scan is desirable for many applications such as CT-based radiotherapy, and for diagnostic and therapeutic purposes. Additionally, CT-based MR image estimation technique can not only increase the diagnostic value of a CT scan but also provide additional reference information for diagnosis. In our study, we propose a synthetic method based on generative adversarial networks (GANs) [4] which is a type of convolutional neural network (CNN) [5] method to estimate MRI from CT.

Recently, CNN has become popular in medical imaging fields, such as skin cancer classification [6], X-ray organ segmentation [7], brain lesion detection [8], and retinal image synthesis [9]. However, training CNN for the above applications needs a substantial amount of ground-truth data, and medical imaging fields are different from general computer vision community. The annotation work can only be conducted by experts who have enough knowledge about medicine or engage in medically related work. Moreover, freely shared or publicly open datasets are prohibited, considering the personal privacy of patients and strict hospital supervision. Therefore, ground-truth data are considered confidential in the medical imaging fields. For the purpose of this study, we require CT and the corresponding MR images to estimate MR from CT, which we refer to as paired data; however, obtaining these is a big challenge. Patients should undergo both CT scan and MR imaging within a short time interval. Moreover, further post-processing such as image registration is also required. Fortunately, the hospitals have an unlimited number of single CT or single MR data, which we refer to as unpaired data. In this study, the MR-GAN framework is proposed for obtaining accurate synthetic results (estimating MR from CT) simultaneously using the limited paired data and substantial unpaired data. The MR-GAN has two structures—the paired cycle-consistent and unpaired cycle-consistent, to simultaneously train different data. The results of the MR-GAN using paired and unpaired data together indicate a better performance according to the quantitative and qualitative evaluations than using only one type of data. To the best of our knowledge, this is the first study that attempts to estimate an MR image from a CT image using GANs. In this study, a synthetic approach is proposed to produce estimated MR images from brain CT images, using both paired and unpaired data. The major contributions of this study can be summarized as follows:

- The proposed approach uses paired and unpaired data to overcome the context-misalignment issue of unpaired training, and to alleviate the registration and blurry results of paired training.
- The MR-GAN framework is introduced by combining adversarial loss, dual cycle-consistent loss, and voxel-wise loss for training paired and unpaired data together.
- The proposed approach can be easily extended to other data synthesis tasks to benefit the medical image community.

Before describing the proposed algorithm, this paper provides a literature survey on medical image synthesis in Section 2. The MR-GAN is presented in Section 3, which includes four subsections: Section 3.1 introduces a method of data acquisition, and Section 3.2 and Section 3.3 briefly explain the structure of the MR-GAN and its objective functions; Section 3.4 describes the implementation details. Section 4 reports the experimental results. The discussions and conclusions are presented in Section 5 and Section 6, respectively.

## 2. Related Work

Many traditional methods have been proposed for estimating one image domain from another. The existing methods can be divided into three categories: tissue-segmentation-based [10], learning-based [11], and atlas-based methods [12]. The tissue-segmentation-based methods first segment the MR image voxels into different tissue classes, such as air, fat, soft tissue, and bone, and then refine the segmentation classes manually. Learning-based methods extract features to represent two different domains and then construct a nonlinear mapping between them. Atlas-based methods apply image registration to align an MR image to an atlas MR image for approximating a correspondence matrix. The matrix can then be used to warp the associated atlas CT image to estimate the query MR image.

Advances in deep learning and machine learning in medical computer-aided diagnosis (CAD) [13,14] have allowed systems to provide information on potential abnormalities in medical imaging. Many methods have synthesized a CT image from the available MR image for MR-only radiotherapy treatment planning [15]. The MR-based synthetic CT generation method [16] used deep convolutional neural networks (CNN) with paired data, which was rigidly aligned with the minimization of voxel-wise differences between CT and MR images. Zhao et al. [17] also proposed a similar method, using a modified U-Net [18] to synthesize an MR image from a CT image, and then utilizing the synthetic MR image for CT-based brain segmentation. However, minimizing the voxel-wise loss between the synthesized image and the reference image during training may lead to blurry generated outputs. To obtain clear results, Nie et al. [19] proposed a method that combined the voxel-wise loss with an adversarial loss in a generative adversarial network (GAN). A concurrent work [20] proposed a similar method to synthesize positron emission tomography (PET) images from CT images using multiple channel information of the pixel-to-pixel (pix2pix) framework by Isola et al. [21]. Ben-Cohen et al. [22] combined a fully convolutional network (FCN) [23] and the pix2pix [21] model to export initial results and blend the two outputs to generate a synthesized PET image from a CT image. Although the combination of voxel-wise loss with adversarial loss addresses the problem of blurry generated synthesis, the voxel-wise loss is dependent on the availability of a large numbers of aligned CT and MR images. Moreover, obtaining rigidly aligned data can be difficult and expensive.

**Figure 1:**
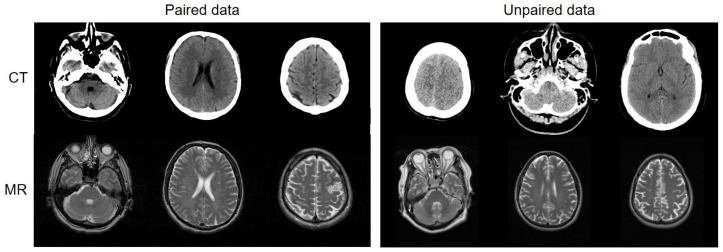
(**Left**): Deep networks train with paired data, which include CT and MR slices taken from the same patient at the same anatomical location. Paired data must be intentionally collected and aligned, which imposes difficulty. However, paired data provide network regression constraints that are far more correct. (**Right**): Deep networks train with unpaired data, which include CT and MR slices taken from different patients at different anatomical locations. There is a considerable amount of unpaired data available.

Most medical institutions have considerable unpaired data that were scanned for different purposes and different radiotherapy treatments. Being able to use the unpaired data would increase the amount of available training data exponentially and alleviate many of the constraints of the existing deep learning-based synthetic systems (Figure 1). Unlike the paired-data-based methods in [16,19,20,22], Wolterink et al. [24] used a CycleGAN model [25], which is an image-to-image translation that uses unpaired images to synthesize CT images from MR images. In an unpaired GAN paradigm, it is desirable for the synthesized image to look real and be paired up with an input image in a meaningful way. Therefore, cycle-consistency loss is enforced to translate the synthesized image back to the original image domain and minimize the difference between the input and the reconstructed image as a regularization. Because of a large amount of unpaired data, the synthesized images are more realistic than the results from the paired training methods. However, compared to the voxel-wise loss of the paired data, the cycle-consistent loss still has certain limitations in correctly estimating the contextual information of soft tissues and blood vessels. Therefore, in our study, the objective function of the MR-GAN includes voxel-wise loss and cycle-consistent loss to benefit from the stable optimization of supervised learning and a massive dataset of unsupervised learning.

## 3. Materials and Methods

### 3.1. Data Acquisition

**Figure 2:**
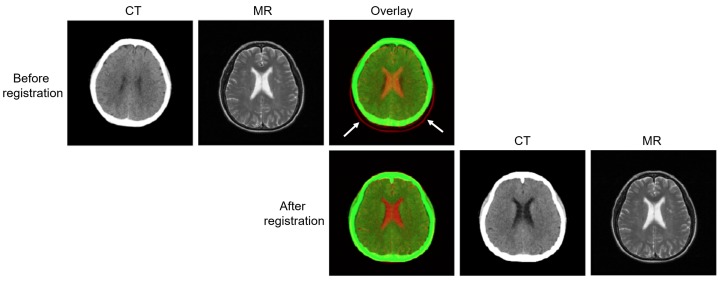
Examples showing registration between CT and MR images after the mutual-information affine transform.

Dataset used in this study was evaluated and approved by the Institutional Review Board (IRB) of the Pusan National University Hospital, South Korea (IRB No. 1808-008-069). The IRB is organized and operates according to the International Council of Harmonization (ICH), Guideline for Good Clinical Practice (GCP), and the applicable laws and regulations. Our dataset consisted of the brain CT and MR images of 202 patients who were scanned for radiotherapy treatment planning for brain tumors. Among these, 98 patients had only CT images and 84 had only MR images; these were the unpaired data. For the remaining 20 patients, both CT and MR images were acquired during the radiation treatment. The CT images were acquired helically on a GE Revolution CT scanner (GE Healthcare, Chicago, IL, USA) at 120 kVp and 450 mA. The T2 3D MR (repetition time = 4320 ms; echo time = 95 ms; flip angle = 150°) images were obtained using a Siemens 3.0T Trio TIM MR scanner (Siemens, Erlangen, Germany). To generate the paired sets, the CT and MR images of the same patient were aligned and registered using affine transformation based on mutual information; the images were resampled to the same voxel size $\left(1.00\times 1.00\times 1.00\;{\mathrm{mm}}^{3}\right)$. Before the registration, the skull area in the CT images was removed by masking all voxels above a manually selected threshold. The skull-stripped MR brain images were also registered. In this study, AFNI(Analysis of Functional NeuroImages)’s 3dAlleniate function was used for the regression process [26]. The affine transformation parameters obtained were used to register the resampled CT and MR images with the skull. To maximize information inside the brain area, the CT images were windowed with a window length of 80 Hounsfield units (HU) and a window center of 40 HU. After registration (Figure 2), the CT and MR images were well-aligned spatially and temporally.

### 3.2. MR-GAN

**Figure 3:**
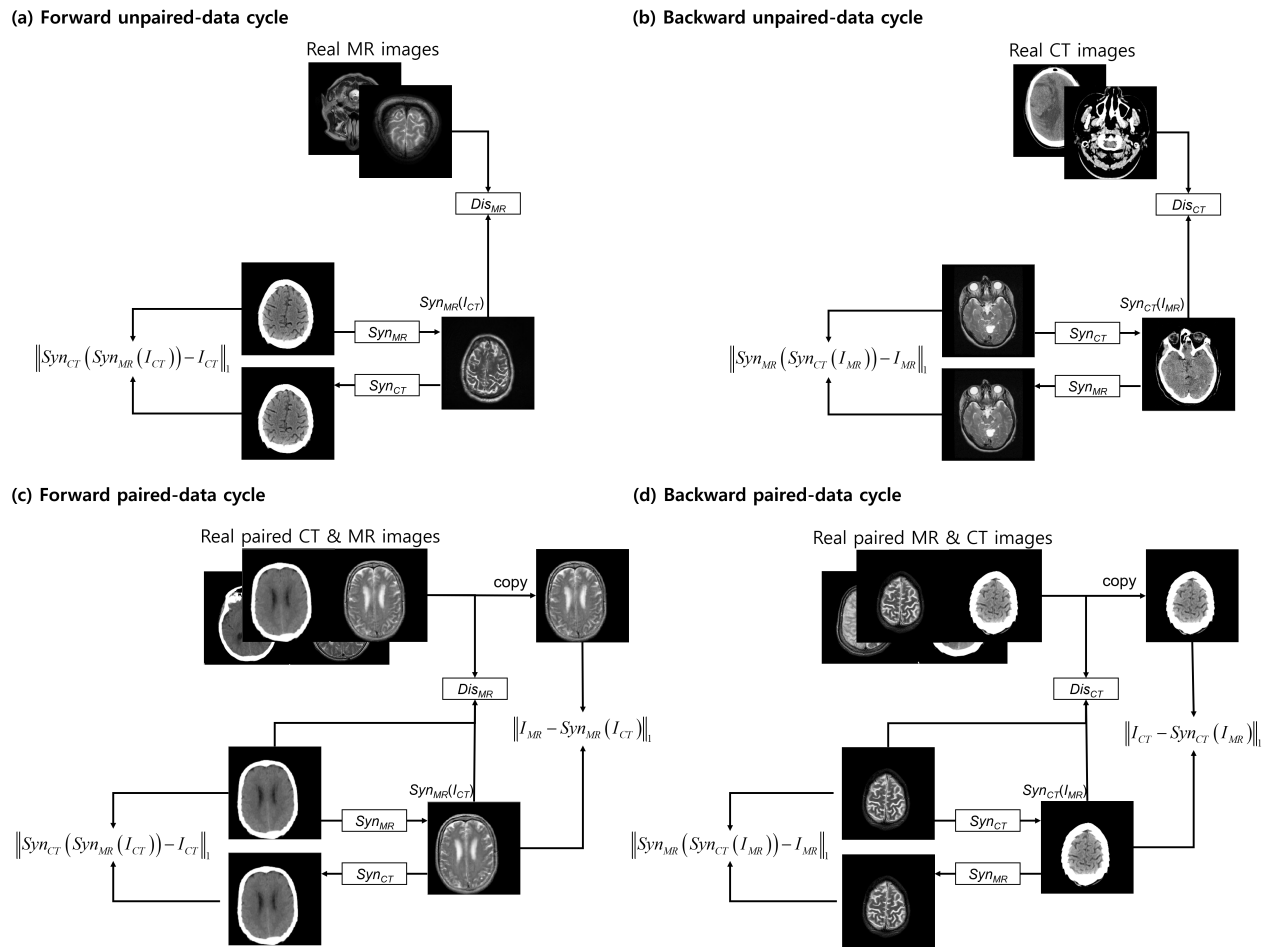
Dual cycle-consistent structure consists of: (**a**) a forward unpaired-data cycle; (**b**) a backward unpaired-data cycle; (**c**) a forward paired-data cycle; and (**d**) a backward paired-data cycle.

The proposed approach has a structure similar to that of the CycleGAN [25], which contains a forward and a backward cycle. However, our model has a dual cycle-consistent term for paired and unpaired training data. The dual cycle-consistent term includes four cycles: forward unpaired-data, backward unpaired-data, forward paired-data, and backward paired-data cycles (Figure 3).

The forward unpaired-data cycle contains three independent networks, each with a different goal. The network $Syn_{\;M\;R}$ attempts to estimate a realistic MR image using a CT image $I_{CT}$ such that the output cannot be distinguished from the “real” MR images by the adversarial-based trained discriminator $Dis_{\;M\;R}$, which is trained to discriminate the synthetic “fakes”. To solve the well-known problem of mode collapse, the network $Syn_{CT}$ is trained to estimate $Syn_{\;M\;R}\left(I_{CT}\right)$ similar to the original CT domain. To improve training stability, the backward unpaired-data cycle is also enforced in estimating a CT image using an MR image. It works with a logic that is opposite to that of the forward unpaired-data cycle.

In the forward unpaired-data cycle, an MR image is approximated from the input CT image by a synthesis network $Syn_{\;M\;R}$. The synthesized MR image is converted to a CT image that approximates the original CT image. $Dis_{\;M\;R}$ is trained to distinguish between the real and synthesized MR images. In the backward unpaired-data cycle, on the other hand, a CT image is synthesized from an input MR by the network $Syn_{CT}$; $Syn_{CT}$ reconstructs the MR from the synthesized CT image and $Dis_{CT}$ is trained to distinguish between the real and synthesized CT images.

The forward and the backward paired-data cycles are the same as the abovementioned forward and backward unpaired-data cycle. However, unlike the unpaired-data cycles, the discriminators in the paired-data cycles not only discriminate between real and synthesized images; they also observe a pair of CT and MR images to differentiate between the real and synthesized pairs. In addition, the voxel-wise loss between the synthesized and the reference images is included in the paired-data cycles. The synthesis networks $Syn_{\;M\;R}$ and $Syn_{CT}$ in the paired-data cycles work exactly in the same manner as those in the unpaired-data cycles.

### 3.3. Objective Function

Both networks in GAN were trained simultaneously with the discriminators $Dis_{\;M\;R}$ and $Dis_{CT}$, estimating the probability that a sample came from real data rather than from the synthesis networks, while the synthesis networks $Syn_{\;M\;R}$ and $Syn_{CT}$ were trained to estimate the realistic synthetic data that could not be distinguished from the real data by the discriminators. Adversarial losses [4] were applied to the synthesis network $Syn_{\;M\;R}$: $I_{CT}\to I_{\;M\;R}$ and its discriminator $Dis_{\;M\;R}$. The objective is expressed as follows:

(1) $$\begin{array}{cc} L_{G\;A\;N}\left(Syn_{\;M\;R},Dis_{\;M\;R},I_{CT},I_{\;M\;R}\right) & =E_{I_{\;M\;R}∼p_{d\;a\;t\;a}\left(I_{\;M\;R}\right)}\left[\log Dis_{\;M\;R}\left(I_{\;M\;R}\right)\right]\\ & +E_{I_{CT}∼p_{data}\left(I_{CT}\right)}\left[\log\left(1-Dis_{\;M\;R}\left(Syn_{\;M\;R}\left(I_{CT}\right)\right)\right)\right]\\ & +E_{I_{CT},I_{\;M\;R}∼p_{data}\left(I_{CT},I_{\;M\;R}\right)}\left[\log Dis_{\;M\;R}\left(I_{CT},I_{\;M\;R}\right)\right]\\ & +E_{I_{CT}∼p_{data}\left(I_{CT}\right)}\left[\log\left(1-Dis_{\;M\;R}\left(I_{CT},Syn_{\;M\;R}\left(I_{CT}\right)\right)\right)\right], \end{array}$$

where $Syn_{\;M\;R}$ attempts to convert an $I_{CT}$ image to a $Syn_{\;M\;R}\left(I_{CT}\right)$ image that looks similar to an image from the MR image domain. In the first and second terms in Equation (1), the discriminator $Dis_{\;M\;R}$ aims to distinguish between the synthesized $Syn_{\;M\;R}\left(I_{CT}\right)$ and reference MR image $I_{\;M\;R}$ for the unpaired data. In the third and fourth terms in Equation (1), the discriminator $Dis_{\;M\;R}$ further attempts to discriminate between the real and synthesized pairs that provide $I_{CT}$ with the synthesized MR image for the paired data. The synthesis network $Syn_{\;M\;R}$ tries to minimize this objective against an adversarial $Dis_{\;M\;R}$ that tries to maximize it, i.e., $Syn_{\;M\;R}^{*}=\arg\min_{Syn_{\;M\;R}}\max_{Dis_{\;M\;R}}L_{G\;A\;N}\left(Syn_{\;M\;R},Dis_{\;M\;R},I_{CT},I_{\;M\;R}\right)$. For another synthesis network $Syn_{CT}$, $I_{\;M\;R}\to I_{CT}$ and discriminator $Dis_{CT}$ have a similar adversarial loss as well, i.e., $Syn_{\;C\;T}^{*}=\arg\min_{Syn_{\;C\;T}}\max_{Dis_{\;C\;T}}L_{G\;A\;N}\left(Syn_{\;C\;T},Dis_{\;C\;T},I_{\;M\;R},I_{CT}\right)$.

To stabilize the training procedure, the negative log-likelihood objective in the unpaired data was replaced by the least-squares loss [27] in this study. Hence, the discriminator $Dis_{\;M\;R}$ aims to apply label 1 for the reference MR images and label 0 for the synthesized MR images. However, it was observed that a negative log-likelihood objective in the paired data generated higher quality results. Equation (1) then becomes:

(2) $$\begin{array}{cc} L_{G\;A\;N}\left(Syn_{\;M\;R},Dis_{\;M\;R},I_{CT},I_{\;M\;R}\right) & =E_{I_{\;M\;R}∼p_{data}\left(I_{\;M\;R}\right)}\left[{\left(Dis_{\;M\;R}\left(I_{\;M\;R}\right)-1\right)}^{2}\right]\\ & +E_{I_{CT}∼p_{data}\left(I_{CT}\right)}\left[Dis_{\;M\;R}{\left(Syn_{\;M\;R}\left(I_{CT}\right)\right)}^{2}\right]\\ & +E_{I_{CT},I_{\;M\;R}∼p_{data}\left(I_{CT},I_{\;M\;R}\right)}\left[\log Dis_{\;M\;R}\left(I_{CT},I_{\;M\;R}\right)\right]\\ & +E_{I_{CT}∼p_{data}\left(I_{CT}\right)}\left[\log\left(1-Dis_{\;M\;R}\left(I_{CT},Syn_{\;M\;R}\left(I_{CT}\right)\right)\right)\right]. \end{array}$$

The dual cycle-consistent loss is enforced to further reduce the space of possible mapping functions for paired and unpaired training data. For each $I_{CT}$ from the CT domain in the forward cycle, the image estimation cycle should be able to bring $I_{CT}$ back to the original image, i.e., $I_{CT}\to Syn_{\;M\;R}\left(I_{CT}\right)\to Syn_{CT}\left(Syn_{\;M\;R}\left(I_{CT}\right)\right)\approx I_{CT}$. Similarly, for each $I_{\;M\;R}$ from the MR domain, $Syn_{CT}$ and $Syn_{\;M\;R}$ should also satisfy a backward cycle consistency: $I_{\;M\;R}\to Syn_{CT}\left(I_{\;M\;R}\right)\to Syn_{\;M\;R}\left(SynCT\left(I_{\;M\;R}\right)\right)\approx I_{\;M\;R}$. The dual cycle-consistency loss is expressed as follows:

(3) $$\begin{array}{cc} L_{dual-cyc}\left(Syn_{\;M\;R},Syn_{CT}\right)= & E_{I_{CT}∼p_{data}\left(I_{CT}\right)}\left[∥Syn_{CT}\left(Syn_{\;M\;R}\left(I_{CT}\right)\right)-I_{CT}{∥}_{1}\right]\\ & +E_{I_{\;M\;R}∼p_{data}\left(I_{\;M\;R}\right)}\left[∥Syn_{\;M\;R}\left(Syn_{CT}\left(I_{\;M\;R}\right)\right)-I_{\;M\;R}{∥}_{1}\right]\\ & +E_{I_{CT},I_{\;M\;R}∼p_{data}\left(I_{CT},I_{\;M\;R}\right)}\left[∥Syn_{CT}\left(Syn_{\;M\;R}\left(I_{CT}\right)\right)-I_{CT}{∥}_{1}\right]\\ & +E_{I_{\;M\;R},I_{CT}∼p_{data}\left(I_{\;M\;R},I_{CT}\right)}\left[∥Syn_{\;M\;R}\left(Syn_{CT}\left(I_{\;M\;R}\right)\right)-I_{\;M\;R}{∥}_{1}\right]. \end{array}$$

Previous approaches [28] have found it beneficial to combine the adversarial loss with a more traditional loss (such as L1 distance). For the paired data $\left\{I_{CT},I_{\;M\;R}\right\}$, the synthesis network $Syn_{\;M\;R}$ is tasked with generating realistic MR images that are close to the reference $I_{\;M\;R}$ of the input $I_{\;C\;T}$. Although a synthesis network $Syn_{CT}$ is not required as a final product, adding the same constraint to the $Syn_{CT}$ enables a higher quality of synthesized MR images. The L1 loss term for the $Syn_{\;M\;R}$ and $Syn_{CT}$ is defined as follows:

(4) $$\begin{array}{cc} L_{L1}\left(Syn_{\;M\;R},Syn_{CT}\right)= & E_{I_{CT},I_{\;M\;R}∼p_{data}\left(I_{CT},I_{\;M\;R}\right)}\left[∥I_{\;M\;R}-Syn_{\;M\;R}\left(I_{CT}\right){∥}_{1}\right]\\ & +E_{I_{\;M\;R},I_{CT}∼p_{data}\left(I_{\;M\;R},I_{CT}\right)}\left[∥I_{CT}-Syn_{CT}\left(I_{\;M\;R}\right){∥}_{1}\right]. \end{array}$$

Therefore, the overall objective is defined as follows:

(5) $$\begin{array}{cc} L\left(Syn_{\;M\;R},Syn_{CT},Dis_{\;M\;R},Dis_{CT}\right)= & L_{\;G\;A\;N}\left(Syn_{\;M\;R},Dis_{\;M\;R},I_{CT},I_{\;M\;R}\right)\\ & +L_{\;G\;A\;N}\left(Syn_{CT},Dis_{CT},I_{\;M\;R},I_{CT}\right)\\ & +\lambda L_{dual-cyc}\left(Syn_{\;M\;R},Syn_{CT}\right)\\ & +\gamma L_{L1}\left(Syn_{\;M\;R},Syn_{CT}\right), \end{array}$$

where λ and γ control the relative importance of the adversarial loss, dual cycle-consistent loss, and voxel-wise loss. We aim to solve the:

(6) $$Syn_{\;M\;R}^{*}=\arg\min_{Syn_{\;M\;R},Syn_{CT}}\max_{Dis_{\;M\;R},Dis_{CT}}L\left(Syn_{\;M\;R},Syn_{CT},Dis_{\;M\;R},Dis_{CT}\right).$$

The MR-GAN procedure is described in Algorithm 1.

**Algorithm 1** MR-GAN, proposed algorithm. All experiments in the paper used the default values m=1, $n_{iter}=1$
**Require:** learning rate α, batch size *m*, number of alternating iterations of the unpaired/paired data $n_{inter}$.1:**for** number of training iterations **do**   2:    **for** $n_{iter}$ steps **do**   3:        Sample ${\left\{I_{CT}^{\left(i\right)}\right\}}_{i=1}^{m}∼P_{data}\left(I_{CT}\right)$ a batch from the unpaired CT data   4:        Sample ${\left\{I_{\;M\;R}^{\left(i\right)}\right\}}_{i=1}^{m}∼P_{data}\left(I_{\;M\;R}\right)$ a batch from the unpaired MR data   5:        Update the discriminator, $Dis_{\;M\;R}$, by descending its stochastic gradient:
$${▽}_{\theta_{d}^{\;M\;R}}\frac{1}{m}\sum_{i=1}^{m}\left[{\left(Dis_{\;M\;R}\left(I_{\;M\;R}^{\left(i\right)}\right)-1\right)}^{2}+Dis_{\;M\;R}{\left(Syn_{\;M\;R}\left(I_{CT}^{\left(i\right)}\right)\right)}^{2}\right]$$6:        Update the generator, $Syn_{\;M\;R}$, by descending its stochastic gradient:
$${▽}_{\theta_{g}^{\;M\;R}}\frac{1}{m}\sum_{i=1}^{m}\left[{\left(Dis_{\;M\;R}\left(Syn_{\;M\;R}\left(I_{CT}^{\left(i\right)}\right)\right)-1\right)}^{2}+∥Syn_{CT}\left(Syn_{\;M\;R}\left(I_{CT}^{\left(i\right)}\right)\right)-I_{CT}^{\left(i\right)}{∥}_{1}\right]$$7:        Update the discriminator, $Dis_{CT}$, by descending its stochastic gradient:
$${▽}_{\theta_{d}^{CT}}\frac{1}{m}\sum_{i=1}^{m}\left[{\left(Dis_{CT}\left(I_{CT}^{\left(i\right)}\right)-1\right)}^{2}+Dis_{CT}{\left(Syn_{CT}\left(I_{\;M\;R}^{\left(i\right)}\right)\right)}^{2}\right]$$8:        Update the generator, $Syn_{CT}$, by descending its stochastic gradient:
$${▽}_{\theta_{g}^{CT}}\frac{1}{m}\sum_{i=1}^{m}\left[{\left(Dis_{CT}\left(Syn_{CT}\left(I_{\;M\;R}^{\left(i\right)}\right)\right)-1\right)}^{2}+∥Syn_{\;M\;R}\left(Syn_{CT}\left(I_{\;M\;R}^{\left(i\right)}\right)\right)-I_{\;M\;R}^{\left(i\right)}{∥}_{1}\right]$$9:    **end for**  10:    **for** $n_{iter}$ steps **do**   11:        Sample ${\left\{I_{CT}^{\left(i\right)},\;I_{\;M\;R}^{\left(i\right)}\right\}}_{i=1}^{m}∼P_{data}\left(I_{CT},I_{\;M\;R}\right)$ a batch from the paired data   12:        Update the discriminator, $Dis_{\;M\;R}$, by ascending its stochastic gradient: $${▽}_{\theta_{d}^{\;M\;R}}\frac{1}{m}\sum_{i=1}^{m}\left[\log Dis_{\;M\;R}\left(I_{CT}^{\left(i\right)},I_{\;M\;R}^{\left(i\right)}\right)+\log\left(1-Dis_{\;M\;R}\left(I_{CT}^{\left(i\right)},Syn_{\;M\;R}\left(I_{CT}^{\left(i\right)}\right)\right)\right)\right]$$13:        Update the generator, $Syn_{\;M\;R}$, by descending its stochastic gradient:
$$\begin{array}{ccc} {▽}_{\theta_{g}^{\;M\;R}}\frac{1}{m}\sum_{i=1}^{m} & \log\left(1-Dis_{\;M\;R}\left(I_{CT}^{\left(i\right)},Syn_{\;M\;R}\left(I_{CT}^{\left(i\right)}\right)\right)\right)+∥Syn_{CT}\left(Syn_{\;M\;R}\left(I_{CT}^{\left(i\right)}\right)\right)-I_{CT}^{\left(i\right)}{∥}_{1} & +∥I_{\;M\;R}^{\left(i\right)}-Syn_{\;M\;R}\left(I_{CT}^{\left(i\right)}\right){∥}_{1} \end{array}$$14:        Update the discriminator, $Dis_{CT}$, by ascending its stochastic gradient:
$${▽}_{\theta_{d}^{CT}}\frac{1}{m}\sum_{i=1}^{m}\left[\log Dis_{CT}\left(I_{\;M\;R}^{\left(i\right)},I_{CT}^{\left(i\right)}\right)+\log\left(1-Dis_{CT}\left(I_{\;M\;R}^{\left(i\right)},Syn_{CT}\left(I_{\;M\;R}^{\left(i\right)}\right)\right)\right)\right]$$15:        Update the generator, $Syn_{CT}$, by descending its stochastic gradient:
$$\begin{array}{ccc} {▽}_{\theta_{g}^{CT}}\frac{1}{m}\sum_{i=1}^{m} & \log\left(1-Dis_{CT}\left(I_{\;M\;R}^{\left(i\right)},Syn_{CT}\left(I_{\;M\;R}^{\left(i\right)}\right)\right)\right)+∥Syn_{\;M\;R}\left(Syn_{CT}\left(I_{\;M\;R}^{\left(i\right)}\right)\right)-I_{\;M\;R}^{\left(i\right)}{∥}_{1} & +∥I_{CT}^{\left(i\right)}-Syn_{CT}\left(I_{\;M\;R}^{\left(i\right)}\right){∥}_{1} \end{array}$$16:    **end for**  17:
**end for**
18:**return** result


### 3.4. Implementation

For the architecture of synthesis networks $Syn_{\;M\;R}$ and $Syn_{CT}$, the architecture from Johnson et al. [29] was utilized. It consists of a 2D fully convolutional network with one convolutional layer, followed by two strided convolutional layers, nine residual blocks [30], two fractionally strided convolutional layers, and one last convolutional layer. Instance normalization [31] and rectified linear unit (ReLU) followed each convolution except at the last convolutional layer. The synthesis network takes a 256×256 input and generates an output image of the same size.

**Figure 4:**
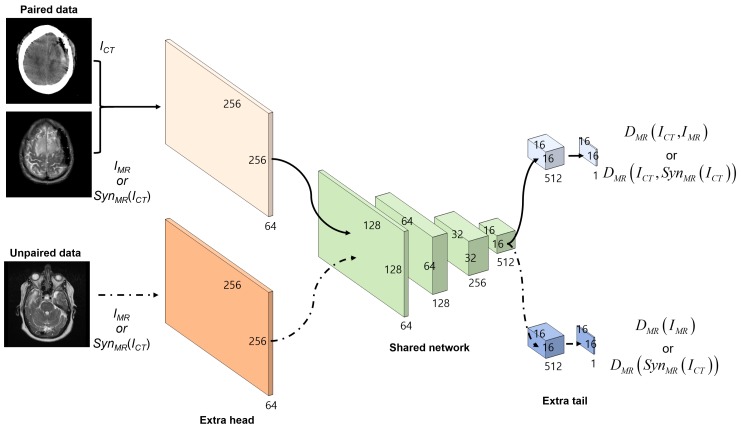
Flow diagram of the discriminator $Dis_{\;M\;R}$ in the synthesis system. $Dis_{\;M\;R}$ has extra head and extra tail convolutional layers for the different input and loss functions of the paired and unpaired data. Discriminator $Dis_{CT}$ has the same architecture as the $Dis_{\;M\;R}$.

For the discriminators $Dis_{\;M\;R}$ and $Dis_{CT}$, the PatchGANs [21] were adapted, which attempt to classify each N×N patch in an image as real or fake. Thus, the discriminators could better focus on the high-frequency information in the local image patches. The $Dis_{\;M\;R}$ and $Dis_{CT}$ networks used the same architecture, which had one convolution as an extra head for different input data, four strided convolutions as a shared network, and two convolutions as an extra tail for different tasks. Except for the first and last convolution, each convolutional layer was followed by instance normalization [31] and leaky ReLU [32] (Figure 4).

To optimize the networks, minibatch stochastic gradient descent (SGD) was used and the Adam optimizer [33] was applied with a batch size of 1. The learning rate started at $2e^{-4}$ for the first $1e^{5}$ iterations and decayed linearly to zero over the next $2e^{5}$ iterations. For all experiments, the following empirical values were used in Equation (5): λ=10 and γ=100. At inference time, only the synthesis network $Syn_{\;M\;R}$ was run to give a CT image.

## 4. Results

### 4.1. Data Preprocessing

Among the data of 202 patients, all the unpaired data were used as training data. The paired data were separated into a training set with the data of 10 patients, a separate test set containing CT images, and the corresponding reference MR images from 10 patients. Each CT or MR volume involved more than 35 2D axial image slices. These were resampled to 256×256 in 256-grayscale and uniformly distributed by HU for CT and MR data. For the training, the training data were augmented with random online transforms:

- *Flip*: Batch data were horizontally flipped with 0.5 probability.
- *Translation*: Batch data were randomly cropped to size 256×256 from padded 286×286.
- *Rotation*: Batch data were rotated by $r\in \left[-5,5\right]$ degrees.

The paired CT and MR images were augmented using the same factor, while the unpaired CT and MR images were augmented independently. The training through the proposed training took about 72 hours for $3e^{5}$ iterations using a single GeForce GTX 1080Ti GPU. At inference time, the system required 35 ms to synthesize a single-slice CT image to MR image. The goal of the adversarial training is to not minimize total loss but to improve the performance of the generator and discriminator during competition. From the experimental observations, we found that $3e^{5}$ iterations were enough to converge the generator and discriminator networks.

### 4.2. Evaluation Metrics

The reference and synthesized MR images were compared using the mean absolute error (MAE), which is defined as follows:

(7) $$M\;A\;E=\frac{1}{N}\sum_{i=0}^{N-1}∥I_{\;M\;R}\left(i\right)-Syn_{\;M\;R}\left(I_{CT}\left(i\right)\right)∥,$$

where *i* is the index of the 2D axial image slice in the aligned voxels and *N* is the number of slices in the reference MR images. MAE measures the average distance between each pixel of the synthesized and the reference MR image. In addition, the synthesized MR images were evaluated using the peak-signal-to-noise-ratio (PSNR) as proposed in [19,20,24]:

(8) $$P\;S\;N\;R=10\cdot \log_{10}\left(\frac{M\;A\;X^{2}}{M\;S\;E}\right),$$

(9) $$M\;S\;E=\frac{1}{N}\sum_{i=0}^{N-1}{\left(I_{\;M\;R}\left(i\right)-Syn_{\;M\;R}\left(I_{CT}\left(i\right)\right)\right)}^{2},$$

where the value of MAX=255. PSNR measures the ratio between the maximum possible intensity value and the mean square error (MSE) of the synthesized and reference MR images. MAE and PSNR are highly dependent on the quality of the image alignment. However, perfect image alignment is always tricky; therefore, the structural similarity (SSIM) index should be also evaluated. It does not merely measure the pixel-wise intensity difference; it also evaluates the luminance, contrast, and structure of the two images, and is defined as follows [34]:

(10) $$S\;S\;I\;M_{i}=\frac{\left(2\mu_{i,x}\cdot \mu_{i,y}+C_{1}\right)\left(2\sigma_{i,xy}+C_{2}\right)}{\left(\mu_{i,x}^{2}+\mu_{i,y}^{2}+C_{1}\right)\left(\sigma_{i,x}^{2}+\sigma_{i,y}^{2}+C_{2}\right)},$$

(11) $$S\;S\;I\;M=\frac{1}{N}\sum_{i=0}^{N-1}S\;S\;I\;M_{i},$$

where $\mu_{i,x}$ and $\mu_{i,y}$ are the average of the *i*th 2D axial image slice of the synthesized and reference MR images; $\sigma_{i,x}^{2}$ and $\sigma_{i,y}^{2}$ represent the variance of the synthesized and reference MR images; $\sigma_{i,xy}$ is the covariance of the synthesized and reference MR images; and $C_{1}$ and $C_{2}$ are two hyper-parameters that stabilize the division with the weak denominator.

### 4.3. Analysis of MR Synthesis Using Paired and Unpaired Data

**Figure 5:**
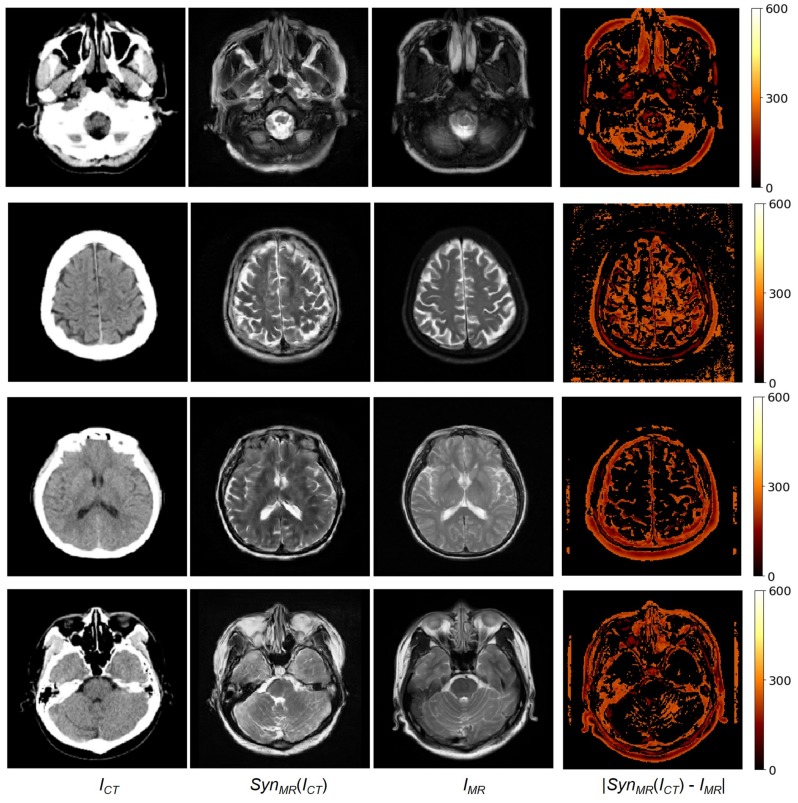
From (**left**) to (**right**): Input CT, synthesized MR, reference MR, and absolute error between reference and synthesized MR images.

Our dataset includes unpaired data of 182 patients and paired data of 20 patients. To train MR-GAN with paired and unpaired data, all the unpaired data were used as training data, and the 20 paired data were equally separated into two groups: 10 pairs for the training set, and the other 10 pairs for the test set. Moreover, one patient’s paired data volume includes about 35 2D axial image slices of CT and MR. There were a total of 366 2D axial image slices in the test set. The same training and test set were used for all the experiments: First, the synthesized MR images were compared with the reference MR images that had been carefully registered to become paired data with CT images. For brevity, the method developed in this study is referred to as the MR-GAN. Figure 5 shows four examples of an input CT image, a synthesized MR image obtained using MR-GAN, a reference MR image, and the absolute difference maps between the synthesized and reference MR images. It is worth mentioning that we reduced the original difference range to $\left[0,600\right]$ to make the absolute difference much clearer for the analysis. The MR-GAN learned to differentiate between different anatomical structures with similar pixel intensity in CT images (such as bones, gyri, and soft brain tissues). The largest differences were found in the area of bony structures, while the smallest differences were found in the soft brain tissues. This may be partly due to the misalignment between the CT and the reference MR images and because the CT image provides more detail about the bony structures to complement the shortcoming of the synthesized MR, which is focused on the soft brain tissues.

Table 1 shows a quantitative evaluation using MAE and PSNR to compare the different methods in the test set. The proposed method is compared with the independent training using the paired and unpaired data. For training the paired data system, a synthesis network with the same architecture as $Syn_{\;M\;R}$ and a discriminator network with the same architecture as $Dis_{\;M\;R}$ were trained using a combination of adversarial loss and voxel-wise loss (as in the pix2pix framework [21]). To train the unpaired data system [24], the cycle-consistent structure of the CycleGAN [25] model was used. This is the same as the approach presented for the forward and backward unpaired-data cycles, shown in Figure 3. To ensure a fair comparison, all the baselines were implemented using the same architecture and implementation details.

**Figure 6:**
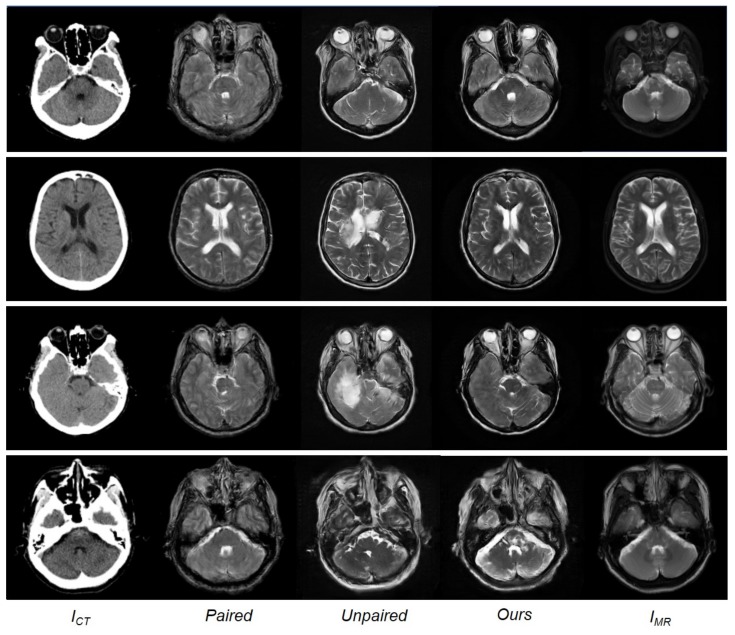
From (**left**) to (**right**): Input CT image, synthesized MR image with paired training, synthesized MR image with unpaired training, synthesized MR images with paired and unpaired training (ours), and the reference MR images.

Although having trained with limited paired data, the model using the paired training data is observed to outperform the CycleGAN model using the unpaired data. Table 1 indicates that the approach adopted in this study, i.e., training with paired and unpaired data together, exhibited the best performance across all measurements, with the lowest MAE and highest PSNR and SSIM values compared to the conventional paired and unpaired training methods. Figure 6 shows a qualitative comparison between the paired training, unpaired training, and the approach presented herein. The results of the training with paired data appeared reasonable but generated blurry outputs. The images obtained with unpaired training were realistic but lost anatomical information in areas of soft brain tissue and contained artifacts in areas with bony structures. The method presented herein learns estimation using paired and unpaired data. While the quality of the results closely approximates the reference MR images, for some details, the results obtained are observed to be much clearer than the reference MR images.

The comparison results for several discriminator models are now presented: as mentioned in Figure 4, the discriminator consists of the extra head, shared network, and extra tail. The different discriminator models are presented in Table 2 using standard 4×4 padded convolution stride 1 with different number of filters. The comparisons of the MAE, PSNR, and SSIM for different discriminator networks and objective functions are provided in Table 3 and Table 4. The results clearly indicate that the discriminators with the negative log-likelihood are better than those with the least squares loss [27] in terms of MAE and PSNR, and SSIM. Furthermore, it is observed that the performance is improved by increasing the number of convolution layers in the extra head and tail of the discriminator. With little convolution layers in the two networks, the discriminator prevents overfitting in the paired and unpaired learning. Note that the performance does not depend on the number of convolution layers in the shared network.

**Figure 7:**
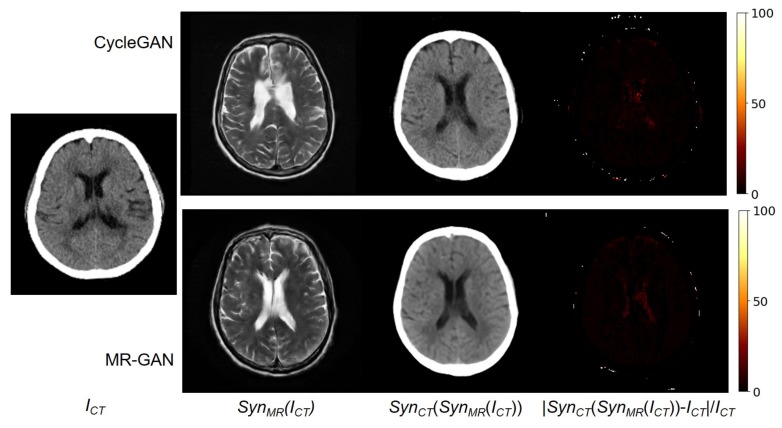
From (**left**) to (**right**): Input CT image, synthesized MR image, reconstructed CT image, and relative difference error between the input and reconstructed CT images.

During the training of the MR-GAN, dual cycle-consistency loss is explicitly imposed in a bidirectional manner. Hence, an input CT image estimated to an MR image by the model should be successfully estimated back to the original CT domain. Figure 7 shows an input CT image, and the corresponding synthesized MR images from the CycleGAN and MR-GAN. It also shows their reconstructed CT images and their relative difference maps. It is observed that the reconstructed CT images are very close to the input images. The relative differences are distributed at the contour of the bone, and the reconstructed CT image from MR-GAN is apparently more smoothed than that from the CycleGAN model. This is because of the correct $Syn_{\;M\;R}\left(I_{CT}\right)$, which is like a latent vector in an auto-encoder [35].

**Figure 8:**
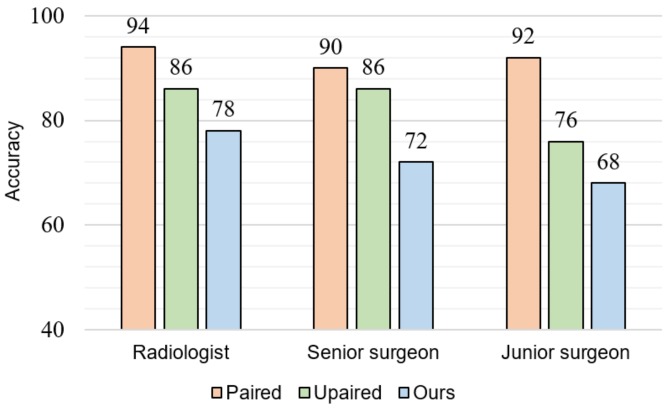
Blind test of “reference vs. estimated” MR images corresponding to the CT images.

To further evaluate whether the reliability of the estimated MR images, the reference and estimated MR images are discriminated by clinicians visually. In addition, the similarities between subdivisions of the reference and estimated MR images are evaluated. Figure 8 indicates blind test results while showing one MR image and one corresponding CT image. Three medical doctors who have never seen the estimated MR images participate in this blind test: one radiologist, one senior surgeon, and one resident. From the test dataset, 50 images are randomly selected and shown to participants. We randomly combine one CT image with its corresponding reference MR image or estimated MR image. The participants classify that they are a reference image pair or estimated image pair. The classification accuracies of our method from three participants are 78%, 72%, and 68%, respectively. This accuracy is significantly lower than the model training with the paired and unpaired data independently, which means that the estimated MR images are realistic enough compared to the reference MR images. When medical doctors are thoroughly confused between the estimated and reference MR images, the classification accuracy would be close to the random guess (50%). The paired method which produces blurry results almost never fooled participants.

Medical doctors also evaluate the qualitative similarity of each structure for the brain MR images in Table 5. The following features are subdivided: hippocampus, caudate, gray matter, white matter, ventricle, corpus callosum, falx cerebri, and overall similarity. For each feature, we randomly select 20 image pairs from the test dataset. The reference MR image, estimated MR image, and corresponding CT image are shown simultaneously. A radiologist and a senior surgeon mark the similarity for each feature from 1 to 10. A higher score represents that the medical experts consider the structure between the estimated and reference MR images are much more similar. Among all features, those with the highest similarity are flax cerebri (8.350), gray matter (8.325), and overall similarity (8.300). In addition, hippocampus (7.450) and ventricle (7.350) have the lowest average similarity due to the small regions occupied and the subtle changes of the Hounsfield unit and small regions in CT scans.

## 5. Discussion

This study has demonstrated that a synthetic system can be trained using paired and unpaired data to synthesize an MR images from a CT image. The proposed approach utilizes the adversarial loss from a discriminator network, dual cycle-consistent loss using unpaired training data, and voxel-wise loss based on paired data to synthesize realistically looking MR images. The quantitative evaluation (results in Table 1) shows that the average correspondence between synthesized and reference MR images in our approach is far superior to the other methods. Specifically, the synthesized images are closer to the reference and achieve the lowest MAE of (19.36), and highest PSNR of (65.35) and SSIM (0.25). However, slight misalignments between the CT images and the reference MR images may have a significant effect on the quantitative evaluation. Although a quantitative measurement may be the gold standard for assessing the performance of a method, in this case, the numerical differences in the quantitative evaluation cannot indicate the qualitative difference correctly. In future works, the accuracy of synthesized MR images based on perceptual studies with medical experts will be evaluated.

Furthermore, the results show that, while a synthetic system using a CycleGAN model trained by the cycle-consistent loss can generate realistic results, they have poor anatomical definitions compared with the corresponding CT images (as exemplified in Figure 6). Moreover, the pix2pix model outperforms the CycleGAN, where pix2pix is trained with the voxel-wise loss. However, it still gets a blurry output because the voxel-wise loss just minimizes the mean square error. Compared with these two methods, the proposed MR-GAN approach can obtain more realistic and less blurring MR images, since we use both voxel-wise loss and adversarial loss together with the proposed dual cycle-consistent loss.

In this study, although the estimated MR images are quantitatively similar to the reference MR images, medical experts still can confidently recognize the estimated MR images. The average classification accuracy of medical experts is 70.27% (Figure 8). For the junior surgeon, the accuracy is less than 70%. Therefore, distinguishing the estimated MR images from MR-GAN by the clinicians with insufficient experience is not an easy task. From the evaluation of the structural similarity in Table 5, we can observe that falx cerebri, gray matter, and corpus callosum are much more similar between the estimated and reference MR images due to their simple structure. In contrast, the structures of hippocampus and ventricle are complicated and occupy a small area in the brain, which cause less similarity between the estimated and reference MR images. However, the radiologists mainly focus on these structures in the brain MR images and may therefore have perceived a high qualitative difference, despite the high quantitative similarity.

A complete workflow based solely on CT can further eliminate image registration uncertainties for combining CT with MRI, and reduce clinical workload. The experimental results have implications for accurate diagnostic purposes and CT-based radiotherapy treatment [1] for patients who are contraindicated to undergo an MR scan because of cardiac pacemakers or metal implants, and for patients who live in areas with poor medical services. Our synthesis system can be trained using any type of data: paired, unpaired, or both. Moreover, the training data can be exponentially increased by using unpaired data together with paired data, which can alleviate the constraints of deep learning-based medical synthetic systems. In addition, the estimated result using paired and unpaired data together has a higher quantitative and qualitative evaluation than using just one type of data.

## 6. Conclusions

A synthetic system using deep generative adversarial networks is presented herein for synthesizing MR images from CT images. The proposed approach named MR-GAN uses paired and unpaired data together to solve the context-misalignment problem of unpaired training and to alleviate the rigid registration task and blurred results of paired training. Because a substantial amount of unpaired data is readily available, it can be used together with the limited paired data for active synthesis in many cases. Results from the test set demonstrate that MR-GAN is much closer to the reference MR images when compared with the other methods. Furthermore, the results indicate that the synthetic system can efficiently estimate structures within the complicated 2D brain slices (such as soft brain vessels, gyri, and bones). The proposed MR-GAN can be applied in CT-based radiotherapy planning by further eliminating image registration uncertainties for combining MRI with CT and reducing clinical workload. In addition, it can also increase the diagnostic value of a CT scan and provide additional reference information for diagnostic purposes. Furthermore, our MR-GAN can be trained by limited paired data and the substantial amount of unpaired data together, which means that our approach can alleviate the labeled data problem for deep learning because unpaired data are largely unlabeled data, which are easy to obtain. Moreover, this approach can also be extended to support other applications such as MR-CT and CT-PET synthesis.

References

## Figures and Tables

**Table 1:** MAE, PSNR, and SSIM evaluations between synthesized and reference MR images when training with paired, unpaired, and MR-GAN using paired and unpaired data together (Ours).

	MAE			PSNR			SSIM		
	Paired	Unpaired	Ours	Paired	Unpaired	Ours	Paired	Unpaired	Ours
Pat-01	24.20	27.71	22.76	62.82	62.45	64.65	0.24	0.25	0.23
Pat-02	17.82	24.12	18.27	64.91	63.05	65.93	0.24	0.24	0.25
Pat-03	22.01	22.45	22.27	63.59	63.83	63.55	0.25	0.22	0.25
Pat-04	18.23	23.64	16.75	65.28	63.44	65.76	0.24	0.20	0.25
Pat-05	18.26	22.82	17.68	64.92	64.04	65.97	0.22	0.23	0.24
Pat-06	20.52	20.41	17.57	64.87	64.78	65.92	0.22	0.22	0.22
Pat-07	20.63	18.72	16.55	64.55	64.14	66.28	0.23	0.25	0.23
Pat-08	19.42	22.77	18.30	64.10	63.22	65.82	0.22	0.24	0.26
Pat-09	19.12	16.98	18.57	64.93	66.19	65.43	0.25	0.20	0.27
Pat-10	23.23	29.76	24.91	63.81	62.60	64.17	0.25	0.19	0.25
Average	20.34	22.94	19.36	64.28	63.77	65.35	0.24	0.22	0.25

**Table 2:** Different network architectures for the discriminator.

Discriminator	D1	D2	D3	D4	D5
Extra head	(64)	(64, 64)	(64, 64)	(64)	(64)
Shared network	(64, 128, 256, 512)	(64, 128, 256, 512)	(64, 128, 256, 512)	(128, 256, 512)	(128, 256, 512)
Extra tail	(512, 1)	(512, 512, 1)	(512, 512, 512, 1)	(512, 1)	(1)

**Table 3:** Comparison of the MAE, PSNR, and SSIM for different discriminator networks and least squares loss. The leading scores are displayed in bold fonts.

Model	Least Squares Loss		
	D1	D2	D3
MAE (Avg ± sd)	**21.07 ± 2.98**	42.95 ± 3.03	37.25 ± 2.58
PSNR (Avg ± sd)	**65.25 ± 0.81**	61.31 ± 0.64	62.73 ± 0.77
SSIM (Avg ± sd)	**0.24 ± 0.01**	0.21 ± 0.02	0.22 ± 0.01

**Table 4:** Comparison of the MAE, PSNR, and SSIM for different discriminator networks and negative log-likelihood. The leading scores are displayed in bold fonts.

Model	Negative Log-Likelihood				
	D1	D2	D3	D4	D5
MAE (Avg ± sd)	**19.36 ± 2.73**	49.70 ± 3.10	59.06 ± 3.27	**19.35 ± 2.56**	20.57 ± 2.82
PSNR (Avg ± sd)	**65.35 ± 0.86**	60.34 ± 0.60	59.23 ± 0.46	**65.24 ± 0.77**	65.16 ± 0.85
SSIM (Avg ± sd)	**0.25 ± 0.02**	0.21 ± 0.02	0.20 ± 0.02	**0.25 ± 0.01**	0.24 ± 0.01

**Table 5:** Qualitative similarity between the reference and estimated MR images. One radiologist and one senior surgeon evaluate the similarity. The radiologist is abbreviated as R, and the senior surgeon is abbreviated as S.

	Hippo- Campus		Caudate		Gray Matter		White Matter		Ventricle		Corpus Callosum		Falx Cerebri		Overall	
	R	S	R	S	R	S	R	S	R	S	R	S	R	S	R	S
01	6	7	7	7	8	8	7	7	8	8	9	8	7	7	8	8
02	6	9	9	7	8	9	8	7	9	8	9	7	9	8	9	9
03	9	7	9	9	9	9	8	9	9	9	9	9	9	9	8	7
04	8	7	6	7	8	7	9	9	7	7	8	7	8	8	9	8
05	7	7	8	7	10	9	7	8	8	8	9	9	9	9	7	8
06	7	8	6	10	9	9	7	7	6	4	7	9	9	9	7	9
07	6	9	6	9	7	9	9	8	9	9	7	7	9	9	9	10
08	6	9	6	7	7	9	7	8	6	9	9	8	9	9	10	8
09	5	7	9	9	7	9	8	8	7	8	7	9	9	8	8	8
10	9	8	8	9	8	8	9	7	7	9	9	9	9	9	8	9
11	8	7	9	9	8	8	9	9	7	7	9	9	9	9	9	8
12	5	8	9	9	9	7	8	9	6	6	9	8	7	8	7	7
13	8	7	9	7	9	10	9	8	8	8	7	9	9	8	8	9
14	7	8	9	10	9	9	7	7	8	8	7	7	9	8	8	10
15	7	8	9	7	8	9	8	8	7	6	6	5	8	9	9	10
16	7	8	10	9	9	7	9	8	7	6	8	7	8	8	7	7
17	7	7	9	9	8	8	9	7	9	8	9	9	9	8	9	9
18	9	8	6	7	8	9	8	9	7	7	8	8	9	9	8	9
19	7	9	9	9	8	7	9	9	3	6	9	8	7	9	7	7
20	7	9	7	8	7	9	9	7	8	7	9	8	5	6	9	8
Avg.	7.05	7.85	8	8.25	8.2	8.45	8.2	7.95	7.3	7.4	8.2	8	8.35	8.35	8.2	8.4
	7.450		8.125		8.325		8.075		7.350		8.100		8.350		8.300	
